# Potentially Inappropriate Medications Use among Older Adults with Comorbid Diabetes and Hypertension in an Ambulatory Care Setting

**DOI:** 10.1155/2022/1591511

**Published:** 2022-05-09

**Authors:** Monira Alwhaibi

**Affiliations:** ^1^Department of Clinical Pharmacy, College of Pharmacy, King Saud University, Riyadh, Saudi Arabia; ^2^Medication Safety Research Chair, College of Pharmacy, King Saud University, Riyadh, Saudi Arabia

## Abstract

**Objective:**

This study aims to estimate the prevalence of PIMs use and its associated factors among older adults with comorbid diabetes and hypertension.

**Methods:**

A cross-sectional retrospective study was used, including 1,853 older adults (age ≥65 years) with diabetes and hypertension who visited an ambulatory care setting. The study objectives were to estimate the prevalence and factors associated with PIMs use based on the 2019 American Geriatric Society (AGS) Beers criteria.

**Results:**

Almost one out of two individuals had PIMs used, with the average number of medications taken being seven. The most commonly prescribed PIMs were the use of gastrointestinal and endocrine medications. High risk of PIMs use was among those with ischemic heart disease, anxiety, and polypharmacy.

**Conclusions:**

Given the higher PIMs use among older adults with diabetes and hypertension comorbidities, tailored strategies and interventions to minimize PIMs use in this population are warranted.

## 1. Background

Comorbid chronic conditions among the older adult population present many challenges to the health-care system, given the growing prevalence and burden of chronic illnesses. The coexistence of two or more chronic conditions, also known as multimorbidity, is common among older adults [1]. The most common disease cluster is diabetes and hypertension [2]; approximately two-thirds of adults with diabetes have hypertension comorbidities [3]. Managing older adults with multiple chronic diseases is much more complicated than managing those with a single condition, resulting in a complex treatment regime in terms of drug-disease interactions [4]. Clinical guidelines have been developed to describe standards of care to improve the quality of health care. However, most clinical guidelines focus on single diseases and do not always provide management for individuals with comorbidities [5]. Therefore, older adults with comorbidities are at a greater risk of potentially inappropriate medication use due to multiple drugs used to manage their chronic conditions.

Potentially inappropriate medications (PIMs) are defined as medications that should be avoided among older adults due to their risk offset their benefits. Beers criteria are commonly used to identify PIMs; the latest update was in 2019 [6]. Approximately one-third of older adults prescribed at least one potentially inappropriate medication [7–9]. PIMs use imposes a risk of higher health-care expenditure and utilization, adverse drug events, and other negative health outcomes [10–15]. Besides, PIMs use is associated with an economic burden on the patient, payer, and health-care system [7, 16]. The burden can be amplified in older adults with comorbid diabetes and hypertension, which are among the top expensive health conditions [17], and a great economic burden exists when these conditions are comorbid [18].

Despite the growing body of research that has been done among older adults to identify PIMs use, insight into PIMs use among specific disease clusters is limited. Thus, identifying PIMs use among older adults with comorbidities can provide effective clinical setting tools to identify individuals at higher risk than others. Likewise, PIMs identification can improve understanding the prevalence and risk factors of PIMs use among older adults with comorbidities and develop strategies for avoiding and limiting the burden of inappropriate medications. Recent years have witnessed a wide use of real-world data, electronic health records (EHRs), to conduct research and answer practical questions that help health-care providers and policymakers make informed health-care decisions. Using the EHRs, a comprehensive source of inpatient and outpatient health records, the objective of this study was to (1) estimate the prevalence of PIMs use among older adults focusing on diabetes and hypertension comorbidities and (2) identify the factors associated with PIMs use based on the 2019 American Geriatric Society (AGS) Beers criteria [6]. Beers criteria aimed to reduce older adults' drug-related problems comprising exposure to potentially inappropriate medications, drug-drug interactions, drug-disease interactions, and medications that should be used with caution in the older adult. Hypertension and diabetes comorbidities are selected as they are the most common disease clusters and impose a higher burden on patients, payers, and the health-care system.

## 2. Methods

### 2.1. Study Design

A cross-sectional, retrospective study design was conducted. The institutional review board approved the study under protocol number (E-17-2580).

### 2.2. Data Source

This study used data from the electronic health record database. The EHR database is composed of a demographic file, clinical diagnosis file, and prescription drug file. The demographic file provides information about the patients' date of birth, gender, nationality, and encounter type (outpatient or inpatient). The clinical diagnosis file provided information about the health conditions diagnosis (using the International Classification of Diseases–9th edition, Clinical Modification (ICD-9-CM) codes) and the date of clinical diagnosis. The prescription drug file has data about the prescribed medications used. The Institutional Review Board (IRB) approved the study.

### 2.3. Study Population and Setting

The study inclusion criteria were older patients aged 65 years and older with a clinical diagnosis of both diabetes and hypertension that were identified over one year (1 January 2019 to 1 January 2020) from the EHRs and were included in this study. The exclusion criteria were adults <65 years old and those without a clinical diagnosis of both diabetes and hypertension. Diabetic and hypertensive patients were identified using the ICD-9-CM clinical diagnosis codes. Diabetes includes either type I or type II diabetes. The study was conducted among patients who received their care in an ambulatory care setting (i.e., outpatient setting) in a large hospital in the central region of Saudi Arabia. This hospital provides health services at no cost to Saudi citizens, mostly residents in Riyadh, the capital city, and serves as a referral center for the whole country.

### 2.4. Measurements

The updated 2019 American Geriatric Society (AGS) Beers criteria were used to identify PIMs use [6].

This study identified the presence of PIMs use (use of one or more PIMs) by referencing the Beers criteria list. PIMs use was further classified into one, two, and three or more PIMs.

Demographic variables included age, gender, nationality, and marital status. Information about the diagnosed chronic health conditions was identified using the ICD-9-CM codes. This study identified the following chronic conditions: dyslipidemia, ischemic heart disease (IHD), asthma, osteoarthritis, osteoporosis, and anxiety. These conditions were selected as they are highly prevalent among older adults with diabetes and hypertension. Polypharmacy among older adults was measured as the use of five or more medications.

### 2.5. Statistical Analysis

Descriptive and inferential statistics were used to identify the prevalence and associated factors of PIMs use in older adults. Chi-square tests were used to assess the difference between older adults with and without PIMs regarding sociodemographic and clinical characteristics. All factors with a probability value of <0.05 were included in the regression analysis. Binary logistic regression was used to examine the factors associated with a higher likelihood of PIMs use. All statistical tests were performed using the Statistical Analysis Software, version 9.2 (SAS Institute Inc., Cary, NC).

## 3. Results

Table 1 displays the demographic characteristics and health conditions of the study population. There were 1,853 older adults (age ≥65year) with comorbid diabetes and hypertension with an average age of seventy two. Approximately 62% of the study population was women, and the average number of diagnosed coexisting chronic conditions was three. Nearly 64%, 11%, 10%, and 7% of the study population were diagnosed with dyslipidemia, asthma, osteoarthritis, and anxiety, respectively.

This study indicates that PIMs use occurred in 56% of older adults with comorbid diabetes and hypertension (Table 2). In addition, 40%, 13%, and 3% were prescribed one, two, and three or more PIMs, respectively. The most common PIMs used were gastrointestinal medications (54%), followed by endocrine agents (28%). The use of PIMs was significantly higher among those with IHD, anxiety disorder, and those who were taking five or more medications (i.e., polypharmacy) (*p* < .001). Older adults who were taking five or more medications were more likely to have PIMs use (adjusted odds ratio (AOR) = 4.14; confidence interval (CI): 3.06–5.60; *p* < 0.001) compared to those with four or fewer medications (Table 3). PIMs use was more likely among older adults with comorbid IHD (AOR = 2.12; CI: 1.35–3.32; *p* < 0.001) and anxiety (AOR = 3.08; CI: 1.87–5.07; *p* < 0.001) compared to older adults without these comorbidities.

## 4. Discussion

This study found higher use of potentially inappropriate medications among older adults with comorbid diabetes and hypertension when safer alternatives exist. This population is most vulnerable as they suffer from other coexisting chronic conditions and take multiple medications to manage these conditions. The updated 2019 Beers criterion was used to examine PIMs use. Findings from this study indicate that one out of two older adults with comorbid diabetes and hypertension are taking at least one inappropriate medication. Bazargan et al., in their cross-sectional study among 193 older adults with hypertension, found that one out of two participants had inappropriate medication use [19]. Published studies among older adults in the outpatient setting reported that about one-third to two-thirds of older adults are prescribed PIMs [7–9, 20–23]. The rate of PIMs use in this study is considerably higher than the previously reported PIMs use among older adults. The higher rate of PIMs use may reflect the fact that this study focused on older adults with the most common disease cluster, diabetes, and hypertension. These comorbidities usually require the use of multiple medications to manage their conditions. It has to be noted that a commonly prescribed PIMs in the present study was the use of endocrine agents. Given that several drugs to manage diabetes are listed in Beers' criteria, finding a higher rate of inappropriate endocrine medication use was not surprising. Other commonly prescribed PIMs were the use of gastrointestinal agents that include mainly proton pump inhibitors (PPIs); this is consistent with previous studies examining PIMs use among older patients [7, 19, 24].

Furthermore, coexisting chronic conditions were essential factors for PIMs use. Seventy percent of the present study population who had dyslipidemia uses at least one PIM. Moreover, individuals with anxiety and IHD are more likely to use PIMs. Anxiety disorder has been identified as a predictor of PIMs use in other published studies [9, 23, 25]. One of the most likely factors associated with PIMs use in this study was using five or more medications. Indeed, it is not surprising that using multiple medications leads to PIMs use. This finding is consistent with many studies reporting a higher likelihood of using PIMs among older adults using multiple medications [9, 20, 26, 27].

Multiple practical implications can emanate from the present study findings. First, the results can alert prescribers to the potential for improving prescribing in this vulnerable population. Endocrinologists and primary care health-care providers need to provide routine screening for older adults, mainly for individuals taking multiple medications. These screenings can detect PIMs early, thereby preventing the subsequent negative health consequences of inappropriate medications. Wang-Hansen et al., in their study among hospitalized older adults with multimorbidity, found that 44% of the serious adverse drug events could have been prevented by adherence to the screening tool for PIMs [28]. Additionally, a study among geriatric patients admitted to the rehabilitation ward found that decreasing in PIMs use (first-generation antihistamines, antipsychotics, benzodiazepines, and NSAIDs) was correlated with improving rehabilitation health outcomes [29]. Further, stakeholders can incorporate Beers criteria as an indicator to evaluate the quality of prescribing in older adults and support the need for medication therapy management services for older adults with diabetes. There is also a need for increasing awareness of health-care providers of PIMs that should be avoided by older adults, especially those taking care of patients with diabetes and hypertension. A systematic review of twenty-two published studies has shown that one of the barriers for prescribers to stop PIMs includes the knowledge gap and lack of awareness about stopping or changing PIMs [30].

The findings of this study highlighted that the 2019 AGS Beers criteria provide a valuable guide for improving the quality of care for older adults. Given the higher PIMs use among older adults with diabetes and hypertension comorbidities, tailored strategies and interventions to reduce PIMs use in this population are warranted. There is a need for greater vigilance when managing patients with comorbid conditions to avoid inappropriate medications. Medication review and management are essential interventions as PIMs use and polypharmacy are connected, and both are linked to poor health outcomes among older adults. With the projected growth of diabetes and hypertension rates, strategies to minimize avoidable medications among this population are needed. Besides, individual patient's beliefs and values about the use of polypharmacy and PIMs are important before stopping medications.

The risks and benefits of deprescribing drug therapy for patients should be individually evaluated, and the inappropriateness of medications should be defined (e.g., duplication of medications, and drug-drug interactions). Although there is strong evidence for the benefit of deprescribing (i.e., reducing the number of medications or PPIs) from observational studies such as reducing adverse drug reactions, health-care costs, and improving adherence to medications, there are some harms of ceasing medication use such as recurrence of the medical condition and adverse drug withdrawal reactions [31].

### 4.1. Strengths and Limitations

The uniqueness of this study is evaluating the factors affecting PIMs use among older patients with diabetes and hypertension comorbidities, which is a vulnerable population. Electronic health records enabled us to use a large sample size and comprehensive data to identify the prescribed PIMs. However, this study has some limitations. First, this study's findings cannot be generalized to all older adults with comorbid diabetes and hypertension entirely, as this study was conducted in a single setting in Saudi Arabia. Second, we have not adjusted for the severity of diabetes and hypertension and their treatment since this information was not measured. Third, unmeasured confounders such as patients' beliefs and attitudes, prescribers, and health-care system factors were not available in the EHRs and were not adjusted in the analysis.

Furthermore, we have to acknowledge the inherent limitation of explicit tools to identify PMIs, owing to the lack of external validity in Beers criteria, due to differences in regional practices, prescribing patterns, and the availability of medications between practice settings or countries. Therefore, our country should develop a consensus list based on the characteristics of the local population and prescribing practice; in this way, one may have accurate estimates of the prevalence of PIM use. It has to be noted that, however, many of the antihypertensive/cardiovascular drugs listed in Beers criteria should be avoided as first-line therapy; however, we do not have information about whether these drugs are first line or not; therefore, now we have added this critical point to the limitations of the study. Moreover, more details on the type of PIMs medications, the dosage of PIMs, and the duration of administration are essential to measuring the PIMs using Beers criteria; however, these were not collected in this study. Besides, given the nature of the study design, a causal relationship cannot be identified.

## Figures and Tables

**Table 1:** Characteristics of the study population of older adults with comorbid diabetes and hypertension. Number and raw percentage of characteristics by potentially inappropriate medication use.

		Total		PIMs use		No PIMs use			
		*N*	%	*N*	%	*N*	%	*P* value	Sig.
Total		1,853	100	1,039	56	814	44		
Age mean(SD)		72 (6.16)		72 (6.11)		72 (6.25)		0.183	
Average # of medications (SD)		7 (0,19)		8 (2,19)		5 (0,16)		<0.001	∗∗∗
Average # of conditions (SD)		3 (2,8)		3 (2,8)		3 (2,7)		<0.001	∗∗∗
Gender								0.121	
Male		710	38	382	54	328	46		
Female		1,143	62	657	58	486	43		
Marital status								0.086	
Single		71	4	32	45	39	55		
Married		1,573	96	872	55	701	45		
Nationality								0.351	
Saudi		1,728	93	965	56	763	44		
Non-Saudi		123	7	74	60	49	40		
Dyslipidemia								0.124	
Yes		1,181	64	678	57	503	43		
No		672	36	361	54	311	46		
IHD								<0.001	∗∗∗
Yes		128	7	97	76	31	24		
No		1,725	93	942	55	783	45		
Asthma								0.341	
Yes		194	11	115	59	79	41		
No		1,659	90	924	56	735	44		
Osteoarthritis								0.185	
Yes		179	10	92	51	87	49		
No		1,674	90	947	57	727	43		
Osteoporosis								0.692	
Yes		163	9	89	55	74	45		
No		1,690	91	950	56	740	44		
Anxiety								<0.001	∗∗∗
Yes		126	7	99	79	27	21		
No		1,727	93	940	54	787	46		
Depression								0.572	
Yes		26	1	16	62	10	39		
No		1,827	99	1,023	56	804	44		
									
									
Polypharmacy								<0.001	∗∗∗
> =5		1,558	84	957	61	601	39		
0 to 4 drugs		295	16	82	28	213	72		

Note: The study population comprised of 1,853 older adults aged 65 years and older, with comorbid diabetes and hypertension. *T* test was used to assess the association between age and number of medications and PIMs use. IHD: ischemic heart disease; N: number; PIMs: potentially inappropriate medications; Rx: medications; Sig: significance. Asterisks (∗) represent significant differences in PIMs use; ∗∗∗*P* < .001.

**Table 2:** Summary of the findings of potentially inappropriate medications to be avoided for most older adults according to the 2019 Beers criteria.

		*N*	*%*
*Average number of PIMs (SD)*		0.96 (0.86)	
*Average number of medications (SD)*		7.26 (3.16)	
*Potentially inappropriate medications use*			
Yes		1,039	56.1
No		814	43.9
*Number of potentially inappropriate medications*			
No PIM		814	43.9
One PIM		746	40.3
Two PIMs		245	13.2
Three or more PIMs		48	2.6
*Classification of most common PIMs prescribed*			
Gastrointestinal		675	36.43
Endocrine		535	28.87
Pain medications (NSAIDs)		136	7.34
Antidepressants		9	0.49
Antispasmodics		8	0.43
Antipsychotics		4	0.22
Anti-infective		4	0.22
Genitourinary		1	0.05
Anti-Parkinsonian agents		1	0.05

Note: The study population comprised of 1,853 older adults aged 65 years and older, with comorbid diabetes and hypertension. N: number; NSAIDs: nonsteroidal anti-inflammatory drugs; PIMs: potentially inappropriate medications. No use was reported for central or alpha blocker agents, first-generation antihistamines, antithrombotic, barbiturates, benzodiazepines, hypnotics, or skeletal muscle relaxants.

**Table 3:** Adjusted odds ratios and 95% confidence intervals. From logistic regression on PIM use among older patients with comorbid diabetes and hypertension.

		*PIMs use*		
		*AOR*	*95% CI*	Sig.
*Age*		1.01	[0.99-1.02]	
*Gender*				
Male		0.96	[0.77-1.21]	
Female (Ref.)				
*Marital status*				
Single		0.67	[0.40-1.11]	
Married (Ref.)				
*Nationality*				
Saudi		0.79	[0.52-1.20]	
Non-Saudi (Ref.)				
*Dyslipidemia*				
Yes		1.1	[0.88-1.38]	
No (Ref.)				
*IHD*				
Yes		2.12	[1.35-3.32]	∗∗
No (Ref.)				
*Asthma*				
Yes		1.17	[0.83-1.64]	
No (Ref.)				
*Osteoarthritis*				
Yes		0.75	[0.54-1.06]	
No (Ref.)				
*Osteoporosis*				
Yes		0.92	[0.63-1.34]	
No (Ref.)				
*Anxiety*				
Yes		3.08	[1.87-5.07]	∗∗∗
No (Ref.)				
*Depression*				
Yes		0.9	[0.36-2.31]	
No (Ref.)				
*Polypharmacy*				
> =5		4.14	[3.06-5.60]	∗∗∗
0 to 4 drugs (Ref.)				

Note: Study population comprised of 1,853 older adults aged 65 years and older, with comorbid diabetes and hypertension. The reference group for PIMs was “No PIMs use”.

IHD: ischemic heart disease; AOR: adjusted odds ratio; PIMs: potentially inappropriate medications; Ref: reference group; Sig: significance.

Asterisks (∗) represent significant differences in PIMs use.

∗∗∗*P* < .001; ∗∗.001 ≤ *p* < .01.

## Data Availability

The dataset supporting the conclusions of this article is available by request from the corresponding author.
